# 循环肿瘤标志物在肺癌中的应用

**DOI:** 10.3779/j.issn.1009-3419.2015.12.10

**Published:** 2015-12-20

**Authors:** 彩存 周

**Affiliations:** 200433 上海，上海市肺科医院肿瘤科 Department of Oncology, Shanghai Pulmonary Hospital, Shanghai 200433, China

**Keywords:** 液态活检, 循环肿瘤细胞, 循环肿瘤DNA, 靶向PCR, Liquid biopsy, Circulating tumor cells, ctDNA, Ligand-targeted PCR

## Abstract

近年来，“液态活检”概念异军突起，不仅可以辅助诊断某些类型实体肿瘤，而且能够监测复发，评估疗效及肺癌分子表达，更是无创肿瘤筛查方法。其来源主要分为循环肿瘤细胞（circulating tumor cells, CTCs），循环肿瘤DNA（circulating tumor DNA, ctDNA）等。本文讨论了肺癌领域中CTCs、ctDNA及其他肿瘤标志物的生物学特征、检测方法及临床应用，总结了保证特异性前提下的高敏感度、便于临床应用、可重复性好三个重要评判标准的“液态活检”技术，尤其是肺癌Ⅰ期敏感度可达67%的靶向PCR CTC技术的应用，以期使“液态活检”真正从科研探索走向临床应用。

## Abstract (en)

In recent years, "liquid biopsy" received enormous attentionas a new detecting method. As a non-invasive tumor screening method, the applications of liquid biopsyinclude early detection, monitoring relapse, assessment of therapy and molecule expression in lung cancer. The main source of liquid biopsy comes from circulating tumor cells (CTCs), ctDNA, and so on. This review will explore the biological characteristics, detection technologies and clinical applications of CTCs, ctDNA and other tumor markers in lung cancerand summarize liquid biopsy which in accord with three important criteria of high sensitivity (highspecificity), clinical utility and repeatability, especially a new method of ligand-targeted PCR (LT-PCR) that showed a high sensitivity of 67.2% in stage Ⅰ lung cancer. We expect that "liquid biopsy" could be really explored from scientific research to clinical application.

随着分子生物学的快速发展，“精准医疗”已经成为了肿瘤诊疗新进展中最为热门的词汇之一。在肿瘤临床诊疗过程中，特别是肺癌的临床实践中，先明确肿瘤的基因组学类型，是临床治疗的第一步。诸多研究已经证实，表皮生长因子受体（epidermal growth factor receptor, *EGFR*）突变与*ALK*融合基因表达的患者接受相应的靶向治疗的疗效显著优于含铂双药化疗^[1-3]^。因此，国内外的临床指南均推荐肺腺癌患者在接受治疗前必需接受基因检测^[4, 5]^。但是，如果接受基因检测，患者必需接受有创的组织活检，患者所受损伤较大。另一方面，靶向药物通常会在10个月左右产生耐药^[6]^，如何提供有效的手段监测耐药，同时提示耐药机制便成为非常重要的步骤。

基于如上原因，“液态活检”的概念应运而生，有文献^[7]^报道，液态活检是指运用敏感的血液学检测方法在千万背景血细胞中检测并分析潜在的肿瘤细胞。简而言之，就是通过血液检测代替肿瘤组织检测。随着各种高敏感度检测方法的问题，液态活检如果按其来源可分为循环肿瘤细胞（circulating tumor cell, CTC）、循环肿瘤DNA（circulating tumor DNA, ctDNA）、小分子RNA（mircoRNA）、长链非编码RNA（long non-coding RNA）等肿瘤。目前临床常用主要为CTC和ctDNA。

已有较多研究^[8-11]^探索了CTC和ctDNA在肺癌诊疗过程中的应用前景。其中，CellSearch的CTC检测在乳腺癌领域已经有被CFDA批准的检测方法。但是，由于大部分肺癌细胞迁移入血的过程中容易出现上皮间质转化的过程，从而丢失了表面上皮粘附分子的表达。而上皮粘附分子又是CellSearch系统主要的捕获靶标，因此，CellSearch在肺癌中的CTC检测结果并不尽如人意。因此，国内有多种改良技术，在肺癌领域探索了其他方法检测CTC的效率。另一方面，ctDNA检测在国内也处于临床探索的热点。因此，本文主要对于CTC与ctDNA的检测方法、临床应用的侧重点、未来的研究方向进行综述，期望能为临床合理尝试“液态活检”提供依据。

## 1 CTCs

### 1.1 CTCs的前世今生

1869年，澳大利亚一名叫Thomas Ashworth的内科医生^[12]^，他通过显微镜观察到一例死于转移瘤患者，其外周血中存在与原发肿瘤相似的细胞，这样的细胞或许能反映原发肿瘤的特性，被称为CTCs。由于检测手段的限制及细胞的稀少，直到20世纪90年代，CTCs的价值才被内科医生充分认识到。1889年英国病理学家Paget提出了著名的“种子和土壤”假说（seed and soil hypothesis）^[13]^。该假说强调肿瘤细胞和靶组织两个导致肿瘤转移的重要因素，认为器官微环境（“土壤”）可影响特定肿瘤细胞（“种子”）的种植、侵袭、存活、生长，肿瘤转移早期常呈现特异脏器亲合性。其中，CTCs即是该假说中的“种子”，在肿瘤的发生、发展中发挥着重要的作用。此学说成功地阐释了癌症复发和转移的机理。临床研究^[14-16]^显示，CTCs在肺癌的辅助诊断、复发监测、疗效评估及检测肺癌分子表达谱中具有重要的临床意义。

### 1.2 CTCs的生物学特征

肿瘤原发灶或者转移灶中，具有转移倾向一类肿瘤细胞，通过EMT等生物学行为，迁移入血称之为CTC，其可分为具有干细胞特征的CTC和不具有干细胞特征的CTC^[17]^。CTC迁移入血的生物学过程目前尚无明确定论。CTC一旦迁徙入血，其存活时间一般较短，通常不会超过24 h; 而且，就其形态而言，CTC具有高度的异质性^[18]^。因此，即便通过最温和的分离技术，循环还是经常可以检测出已经凋亡或者破坏的CTC。

循环中检测出CTC并不一定意味着患者已经存在远处转移。肿瘤转移是个相对复杂的过程。CTC侵袭入原发灶以外的第二器官（如骨髓等），可称之为播散的肿瘤细胞（disseminated tumor cells, DTC）^[19]^。一般而言，具有干细胞特征的DTC聚集成团，并且形成微转移灶，同时该转移灶可以逃避免疫系统的识别，肿瘤的远处转移才可能发生^[17, 20]^。在肿瘤的浸润和转移过程中，肿瘤细胞会表现出更多的间叶表型特征，失去正常情况下细胞间的相互作用，即存在上皮-间叶转换（epithelial mesenchymal transitios, EMT）过程，从而使其更容易侵入血管内皮而进入血循环^[21]^。在EMT过程中，肿瘤细胞下调了上皮组织特定标记物如角蛋白（cytokeratin, CK）和上皮细胞粘附分子（EpCAM）的表达，上调了间叶组织标记物如波形蛋白的表达^[22, 23]^。但是，EMT并不是一个“全或无”的过程，很多CTCs可以同时表达间叶组织标记物和上皮组织。这对现行的一些CTCs的检测方法有着重要的影响。例如针对上皮组织标记物的检测方法就无法检测到发生EMT的CTCs。

另外一个重要的问题是，早期肿瘤外周循环中是否可以检测到CTC，也就是说CTC迁移入血的过程是发生在肿瘤早期还是晚期？理论上讲，肿瘤大小超过2 mm左右时便可诱导血管生成进入肿瘤，提供血供，从而为肿瘤细胞迁移入血提供基础。Husemann等^[24]^通过研究证实，在转基因小鼠种植后约17-18周，循环中即可检出CK与HER同时阳性表达的细胞，而此时原发肿瘤体积还小于1 mm^3^。另有研究^[25, 26]^结果也再次印证了上述结果，其中He等将表达中等量叶酸受体（folate receptor, FR）的M109鼠肺癌细胞通过皮下注射的方式被移植到BALB/c小鼠的背部侧面。移植2周后，在小鼠耳部的血管中每分钟可以检测到大约1.4个CTC细胞。而到了移植后的第3和4周，则每分钟可分别检测7和18个CTC。随着肿瘤逐渐增大，CTC的数量呈指数级别上升。在肿瘤移植的前4周内，没有在任何组织切片中发现到存在转移性癌症的迹象。因此，理论上而言，在肿瘤发生发展的早期，循环中即可检出CTC。

基于CTC的生物学特征，有学者^[27]^认为，CTC可用于评估肿瘤发生发展的状态与治疗的疗效。且目前的检测方法均是用循环中单位体积血液中CTC的数量用以进一步的分析与评估整体病情。因此，CTC在肺癌领域可以发挥类似肿瘤标志物的作用，用于肺癌的辅助诊断、治疗疗效的评估、术后随访中监测复发。另一方面通过CTC的进一步分析，其亦可用于检测肺癌分子表达谱从而指导临床治疗或者探索肺癌发生发展的分子生物学行为。

### 1.3 CTCs的检测方法

由于CTCs在其外周血中的数量很少，每10^5^-10^7^个有核细胞中才有一个CTCs^[28]^，因此对CTCs的检测技术要求更为准确、敏感。CTCs的检测方法主要分为两部分：CTC富集技术和CTC检测技术。

目前能够检测到外周血中的CTCs的很多方法利用诸如EpCAM、CK等上皮特异性标记物等，但是这些标记物在CTCs中的表达多样，并且存在EMT，因而检测结果可能偏低^[29]^。另外，EpCAM也不是在所有类型的上皮细胞癌中都有表达。通常情况下，血细胞不表达上皮细胞标记物，但一部分白细胞，如巨噬细胞，其CK染色和其他的一些上皮细胞特异性抗原染色却呈阳性^[30]^。因此，同时进行多种上皮细胞标记物阳性染色和白细胞标记物CD45的阴性染色，成为检测CTCs的常规方法。但是，最近有非常多的新型检测方法，已经显示出较为确切的临床价值。

总体而言，CTCs的检测方法很多，而理想的CTC检测方法应该是：高敏感度，易于临床应用和结果可重复（表 1）。

**1:** 肺癌领域CTC检测技术列表 CTC enrichment and detection technologies in lung cancer

	Immunomagnetic beads negative enrichment+ LT-PCR	Immunomagnetic beads positive enrichment+ Immunofluorescence	Immunomagnetic beads negative enrichment+ Immunofluorescence	ISET+ Immunofluorescence
Specificity (%)	88%^[36, 52, 53]^	80%^[54]^	83%^[55-57]^	Unreported
Sensitivity (%)	80%^[36, 52, 53]^	30%^[54]^	Adenocarcinoma: 54%^[57]^	50%^[103]^
Automation	Semi-automatic	Automatic	Semi-automatic	Automatic
Blood volume	3 mL	7.5 mL	4 mL/7.5 mL	10 mL
Sensitivity of stage Ⅰ (%)	67%^[52]^	0%	20% (2/10) ^[55]^	48%^[103]^
Sensitivity of stage Ⅱ, Ⅲ and Ⅳ (%)	89%^[36, 52, 53]^	25%（22/87) ^[54]^	56%-88%^[55, 56]^	50%^[103]^
Visualization	No	Yes	Yes	Yes
CTC: circulating tumor cell; LT-PCR: ligand-targeted PCR.				

#### 1.3.1 CTCs富集技术

CTCs的富集方法主要基于CTCs的物理学特征（大小、密度、电极、可变行性）和生物学特性（细胞表面蛋白、生存能力和侵袭性）进行富集。常用的富集方法有：①密度梯度离心法：利用商品化的分离液，将样品加在惰性梯度介质中进行离心沉降或沉降平衡，根据血液中各种细胞的密度不同，离心后各种细胞成分分层，从下向上依次为：红细胞、中性粒细胞、分离液、单个核细胞（包括CTCs），最上层是血浆，为防止离心前血细胞与分离液混合而不利于分离，建立了OncoQuick方法，用一种专用的50 mL试管，在分离液与血液之间放置多孔屏障，这种方法的局限性是部分肿瘤细胞可迁移至血浆层或在离心管底部形成非特异性的聚合物而丢失CTCs^[31]^。②膜过滤法：2000年主要是利用肿瘤细胞与正常细胞大小不同来分离CTCs的ISET（isolation by size epithelial tumor cells）技术被Vona等^[32]^提出，血液通过孔径8 μm的滤膜过滤，使CTCs聚集于滤膜上，达到富集作用。离心法是在滤膜原理基础上，利用一个内置多孔屏障的专用50 mL试管进行密度梯度离心，避免了全血各个层面的交叉污染，检出率明显高于其他方法^[33]^。此类富集技术简单、方便，可以普遍使用。适合富集任何类型肿瘤的CTCs，富集的CTCs可以继续后续免疫学和基因检测。但由于需要血液标本量大，而且容易出现交叉污染和CTCs的丢失，限制了其进一步应用。过滤法尤其会丢失直径小于滤膜孔径的更有侵袭性的CTC。同时，处理时间过长也是其局限之一。③免疫磁珠分离法：免疫磁珠分离法将抗原抗体反应的高度特异性和免疫磁珠的富集分离作用相结合，达到特异性的生物活性物质和细胞的富集效果，并可对细胞进行形态学分析。从原理上分为阳性（正向）富集和阴性（负向）富集。阳性富集通过肿瘤相关性抗原的抗体直接从外周血中富集得到CTCs，多为基于CTC的某一特征（如表达EpCAM）的正向富集，虽然这类技术分离的CTC纯度较高，但是会造成某些不具有这些特征（如表达EpCAM）的CTC的遗失，让分析从一开始就具有偏向性。阴性富集是通过白细胞相关性抗原CD45等去除白细胞，最大程度的将最易干扰CTC检测的白细胞去除，以此逐步将血液中的血浆、红细胞和白细胞去除，最大程度的保留了稀有的CTC，实现了CTC近100%的回收和富集。上皮细胞通常表达EpCAM、CK和BerEP4，而血细胞不表达，正常血液中亦无上皮细胞，90%的实体瘤来源于上皮细胞，因此，针对EpCAM、CK和BerEP4分子的抗体能用于CTCs的分选。目前CellSearch系统就是基于EpCAM阳性富集得到CTCs，用于临床检测转移性乳腺癌、前列腺癌和结直肠癌患者外周血中的CTCs^[34]^。④微流体芯片（micro-fluidic chip）分离技术：微流体芯片分离技术可以用来处理更大容量的血液标本，可以检测出血液中极微量癌细胞的微流体硅芯片，其表面有数万个包被抗体的微位点，当血液样本流过芯片时，该抗体可与肿瘤细胞结合，因而被粘附在芯片上。目前已经成功开发出第二代机器，为HB-Chip（herringbone-chip）^[35]^，为CTCs更精细的分析奠定了基础。

#### 1.3.2 CTCs检测技术

CTCs的检测也是通过对CTCs特异表达的肿瘤或上皮蛋白或mRNA来进行的。主要方法包括配体靶向PCR法（ligand-targeted PCR method, LT-PCR）、免疫荧光法（immunofluorescence, IF）、流式细胞术（flow cytometry, FCM）、逆转录-聚合酶链反应（reverse-transcriptase PCR, RT-PCR）和酶联免疫斑点法（enzyme-linked epithelial immunospot assay, ELISPOT）。①LT-PCR：该方法是目前最新的CTC检测技术，而且为国内原创。其原理是肺癌等CTC表面会过量表达某些特异性受体，如叶酸受体（folate receptor, FR）^[26]^，靶向PCR利用的配体类似物交联核苷酸片段作为检测探针，通过细胞表面特异性受体与其配体类似物的结合来实现探针与CTC细胞的结合，从而将CTC数目转化为探针的数目，并且通过对探针的PCR定量检测来计算CTC的数目。传统的细胞免疫荧光分析往往对纯度要求很高，负向富集纯度往往满足不了。由于具有受体配体结合（1个CTC表面存在上万个特异性受体）及PCR的两次信号放大，从而可使1个CTC信号放大约10^12^倍。因此，配体介导的靶向PCR法，具有超高的灵敏度的特点，从而使早期肺癌中探测CTC成为了可能。目前，已有多篇文献^[36, 37]^报道了该种方法在肺癌的辅助诊断中的作用。其主要通过FR识别肺癌CTCs。已有发表的组织活检数据^[38-41]^显示约83%的肺癌细胞表面过量表达FR。人FR有三种亚型：FR-α、FR-β和FR-γ。FR-α的表达具有很强的组织和肿瘤特异性，除在少数正常组织（肾、胎盘、脉络丛）有表达之外，在其他正常组织中表达水平非常低。是一种理想的CTCs筛选靶标，保证了特异性。②IF：IF是指以荧光标记的特异性抗体在CTC原位通过抗原抗体反应，可以看见荧光所在的细胞或组织，从而确定抗原或抗体的性质、定位，以及利用定量技术测定含量。该技术的优势在于CTC的鉴定与可视化上，通常而言，认为循环中有核细胞的CK阳性而CD45阴性可鉴定为CTC^[42]^，可伴有或者不伴有其他特殊类型的细胞表面受体阳性（如前列腺癌中的雄激素受体）^[43]^。但是，对于部分但形态学判断为CTC但CK阴性/CD45阴性，或者CK阳性/CD45阴性但形态学上小于白细胞的CTC则需要有经验的医生来判读。另一方面，由于采用荧光显色，1个CTC在显微镜下通常只有一个荧光显像，所以缺乏信号放大的过程，总体而言该方法的特异性较高但敏感性较低。目前国际上最常用的CellSearch CTC检测系统便是使用免疫荧光法，但是目前只被批准适用于转移性的乳腺癌、结直肠癌和前列腺癌的CTCs的检测^[44-46]^。Tanaka等^[47]^有研究证实，其在肺癌领域的敏感性较低，通过CellSearch系统检测101例原发性肺癌患者的CTCs，发现在7.5 mL外周血中，只有32%的Ⅳ期肺癌患者外周血能见到CTCs，IIIa期患者的检出率为0^[48]^。这主要是因为CellSearch系统主要通过上皮表型EpCAM来识别CTCs。而肺癌CTC在迁移入血的过程中通常会发生EMT，从而丢失了上皮细胞特征^[49]^。因此，以上皮表型EpCAM来检测肺癌CTCs的方法，可能会产生假阴性的结果。③FCM：FCM是一种在功能水平上对单细胞或其他生物粒子进行定量分析和分选的检测手段。他可以高速分析上万个细胞，同时从一个细胞中测得多个参数。他利用荧光抗体结合肿瘤细胞使肿瘤细胞染色，然后用流式细胞仪进行分析，还可以对同一个细胞有关物理、化学方面的特性做多参数分析。与传统的荧光镜检查相比，具有速度快、精度高、准确性好等优点。④RT-PCR：RT-PCR的指数扩增是一种很灵敏的技术，是PCR的一种广泛应用的变形，标记组织或肿瘤中某种物质的mRNA作为检测指标，可以检测很低拷贝数的RNA。通过RT-PCR法扩增肿瘤细胞特异性或组织特异性基因，能高度灵敏地从10^6^-10^7^个正常细胞中检测出1个肿瘤细胞，较免疫荧光至少灵敏100倍，是目前检测肿瘤隐匿微转移最有效的方法^[50]^。此方法的局限性在于合适的目的基因选择困难，样本污染容易出现假阳性结果，检测时需要裂解细胞限制了CTCs的进一步使用。⑤ELISPOT：ELISPOT利用活细胞能产生或分泌特异性蛋白的特性，与包埋在培养皿底部的荧光抗体结合，通过检测荧光而间接检测CTCs，这种方法首先应用免疫磁珠分选去除CD45阳性细胞，富集CXCR4（肿瘤转移相关蛋白）阳性的细胞，从而进一步检测分泌特异性蛋白的CTCs。但这种方法只能识别活细胞，因为死亡的细胞不能够分泌足够的蛋白用于检测^[51]^。

### 1.4 CTCs的临床应用

#### 1.4.1 辅助诊断

目前对肺癌的诊断和分期主要基于影像学或病理学检查的结果，对于精确反映肿瘤细胞血循环微转移对患者治疗及预后的影响有一定难度，而CTCs检测则可以弥补这一缺陷。肿瘤转移的重要途径是血液系统，判断临床分期的标准之一是是否发生远处转移。研究结果表明，CTCs与肿瘤分期密切相关，对肺癌诊断具有潜在的应用价值^[47, 59, 60]^。Sher等^[14]^研究发现晚期肺癌患者外周血CTCs阳性率明显高于早期患者，提示结合患者外周血情况，能更准确反映患者的疾病分期和病情发展倾向。如上所述的FR靶向PCR CTC检测技术，通过受试者工作特征曲线（receiver operating characteristic curve，ROC曲线）分析发现，当确定以8.64 CTC Units/3 mL作为cutoff值时，其诊断肺癌的敏感度为80%，特异性为88%。特别值得一提的是，该方法对Ⅰ期NSCLC患者的诊断灵敏度达到67.2%。Ⅱ期、Ⅲ期、Ⅳ期NSCLC患者阳性检出率分别为69.4%、80.9%、100%。与一般肿瘤标志物（NSE、CEA、CYFRA211等）相比，基于FR靶向PCR的CTC检测方法具有更大的曲线下面积（0.823）和约登指数（0.573），且更易去辨别患者是NSCLC还是健康志愿者/肺部良性疾病患者。总体而言，该方法对于肺癌辅助诊断的敏感度（特别是早期肺癌患者的诊断）显著优于目前常用的一般肿瘤标志物^[37]^。

另外，CTCs可在患者出现明显转移前提供预后信息，提高风险评估，并为需要治疗的患者作出指示，所以CTCs检测有可能应用于国际分期系统，作为肿瘤分期的有益补充^[61]^。

#### 1.4.2 复发监测

由于CTC可以评估肿瘤的发生发展状态，而且手术切除原发灶后，血液中CTC数量应该为阴性。因此，理论上而言，在术后监测过程中，CTC数量阳性，可能提示肿瘤存在复发的可能性。

目前仅有少数研究在这方面进行了探索，Sawabata等^[62]^对9例接受肺叶切除术的非小细胞肺癌（non-small cell lung cancer, NSCLC）患者进行了术前术后的CTC检测。结果发现1例患者术前在外周血中检出CTC，而有3例患者在手术后即刻检测中在外周血中发现了CTC，所有患者在术后10 d以后均无法检出CTC。对于CTC在复发监测中的作用，仍需更多研究来探索。

#### 1.4.3 疗效评估

目前的研究显示，CTCs数目和肿瘤的进展、接受药物治疗后的疗效密切相关^[63, 64]^。Punnoose等^[65]^用细胞搜索系统检测了41例患者外周血的CTCs数量，这些患者参加了厄罗替尼及pertuzumab治疗NSCLC的单臂二期临床试验，研究者对其疗效进行氟代脱氧葡萄糖-正电子发射型计算机断层显像（fluorodeoxyglucose-positron emission computed tomography, FDG-PET）和计算机断层扫描（computed tomography, CT）评价，78%的患者在基线水平上可检测到大于1个CTCs，在评估疗效上CTCs数量的减少与FDG-PET影像学检查和按照实体瘤的疗效评价标准结果一致并与较长的疾病进展期（progression-free survival, PFS）相关，CTCs的减少是治疗有效的早期指标。另有一项研究评估了CTC数量波动对化疗疗效的评估作用。研究共检测了25例转移性肺癌患者接受化疗前和化疗2个周期后的CTC数量。结果发现，CTC数量的前后变化与影像学表现一致，CTC下降、无变化、上升患者的无进展生存期分别为2.05个月、3.25个月和8.35个月（*P* < 0.05），提示CTC比一般肿瘤标志物更能反映肿瘤接受化疗后的变化^[56]^。

#### 1.4.4 评估肺癌分子表达

如基因表达分析和全基因组分析等现代基因工程技术正在越来越多地用于提供CTCs分子结构上的信息，已检测到CTC的基因异常与原发灶癌细胞基本一致，包括基因扩增和（或）几种癌基因的等位基因缺失^[66]^、不正常的端粒酶活性^[67]^和非整倍体的变化^[68]^。CTCs上亦可检测到恶性肿瘤的分子足迹^[69]^。如CTCs上各类上皮蛋白质可能为转移提供特异性标记^[70]^如细胞骨架相关细胞因子、信号激酶、生长因子受体或表面粘附分子等的变化。

另外，NSCLC特别是肺腺癌在接受治疗前必需先同时评估*EGFR*与*ALK*基因状态，以此指导后续靶向治疗。虽然，大部分方法检出CTC的敏感度较低，但是一旦富集出CTC，其检测肺癌*EGFR*突变与肿瘤组织对比具有较高的一致性。Maheswaran等^[15]^从20个外周血中检测出CTC的NSCLC患者中，组织与外周血CTC对*EGFR*突变检测的一致率达到了95%，同时亦可以通过检测T790M突变监测患者的耐药。另一项发表在*J Clin Oncol*的研究^[71]^则评估了在CTC中用FISH方法检测*ALK*融合基因的可行性。研究共检测了32例晚期NSCLC患者CTC中*ALK*融合基因表达，其中18例组织学检测为阳性患者中，CTC均为ALK阳性（ALK阳性细胞数大于4个），14例组织学ALK阴性的患者，CTC中ALK阳性细胞表达仅为小于等于1个; 并且，CTC中ALK阳性细胞数可用于评估克唑替尼治疗的疗效。

如上所述，CTC用于肺癌分子基因诊断的不足之处在于CTC本身的检出率低，但就目前的研究结果来看，一旦外周血中富集出足够量的CTC，其基因检测与组织学对比的一致率较高。因此，需要富集效率更高的方法，能够最大程度上完整保留外周血中的CTC，成为此方面研究探索的重要方向。

## 2 ctDNA

血浆游离DNA（cell-free DNA, cfDNA）是外周血中游离存在、不包含在完整细胞结构内的DNA。循环系统中存在cfDNA早已得到证实。目前，cfDNA可能来源于以下三种情况：①来自于细胞的凋亡进程中片段化的DNA; ②来自于坏死的细胞的DNA碎片; ③来自于细胞分泌的exosome。其中，cfDNA主要来源于细胞凋亡。

循环肿瘤DNA（ciuculating tumor DNA, ctDNA）是指肿瘤细胞体细胞DNA经脱落或者当细胞凋亡后释放进入循环系统。ctDNA是人体血液循环系统中不断流动的携带一定特征（包括突变、缺少、插入、重排、拷贝数异常、甲基化等）来自肿瘤基因组的DNA片段。ctDNA就是来源于肿瘤细胞的cfDNA^[72]^，属于cfDNA的一种类型。ctDNA不同于遗传突变的是其来自肿瘤细胞的体细胞突变，而cfDNA存在于体内的每个细胞^[73]^。ctDNA来源：①来自于坏死的肿瘤细胞; ②来自于凋亡的肿瘤细胞; ③CTC; ④来自于肿瘤细胞分泌的外排体^[74]^。

### 2.1 ctDNA分析技术

由于肿瘤特异性的ctDNA并不存在于正常细胞中，他们为癌症检测提供了一种十分敏感和特异的方法。血浆收集技术层面也许会影响ctDNA水平。由于脱氧核糖核酸酶活性，ctDNA在血液中稳定性有限，因此抽血后，包括cfDNA的准备步骤在内不能超出数小时。通常而言，外周血中每毫升血浆中含有17 ng的DNA^[74]^，但其中ctDNA含量低，约占整个循环DNA的1%，甚至只有0.01%^[75, 76]^。检测ctDNA的第一步是抽提外周血中的游离DNA，目前较为通行的抽提方法大约需要1毫升血清或者血浆（3 mL全血，乙二胺四乙酸抗凝），抽血后4 h-5 h内即需要完成抽提步骤。在肺癌领域，ctDNA中检测单个基因突变（如*EGFR*突变）的检测方法较多，如液相色谱法、突变扩增阻滞系统（amplification refractory mutation system, ARMS）法、数字PCR、二代测序法等。总体而言，BEAMing数字PCR法的敏感性最高，可达0.01%，其他方法的敏感性约为1%^[77]^。中国学者在这方面也报道了较多的关键研究。2009年王洁教授有研究^[78]^报道了其用液相色谱法在230例NSCLC患者的外周血中检测*EGFR*突变的研究。结果显示，患者血浆与组织中*EGFR*突变的一致率87%，其中组织*EGFR*突变的77例患者中，63例血浆中检出*EGFR*突变; 组织*EGFR*野生型的153例患者中，137例血浆中检出*EGFR*野生型，血浆与组织的相关系数为0.74。在2015年欧洲肺癌年会公布的IGNITE研究^[79]^中，研究者比较了2, 581例NSCLC患者配对的组织学或者细胞学标本与ctDNA标本的*EGFR*突变吻合度。结果显示，在真实世界中，血液检测较组织学/细胞学检测具有较高的特异性，在亚太地区患者中达到97.2%，说明血液检测的假阳性率低，血液检测*EGFR*基因突变的敏感性为49.6%，血浆与组织的一致率为58%。就上述两篇研究可以看出，ctDNA检测*EGFR*突变的特异性较高，而敏感性较低，因此对于血浆*EGFR*突变阴性结果的解读必需慎重。

就目前而言，对肺癌患者的ctDNA进行多基因甚至是全基因组分析，大部分使用的是二代测序技术。而二代测序技术中，常规的全基因组分析方法由于cfDNA中肿瘤基因组的含量较少，非常容易被大量生殖系基因组学变异（如基因融合与基因拷贝数增加）所埋没。另一方面，如需检测ctDNA中的ALK融合或者ROS1融合，由于包含融合点的DNA序列更为稀少，因此，对二代测序法的测序深度提出了极高的要求^[76, 80, 81]^。这一困难，目前正在被新的二代测序技术所逐渐解决。2014年*Nature Medicine*杂志中，Newman等^[82]^使用一种称之为深度测序肿瘤个体化建档法（cancer personalized profiling by deep sequencing, CAPP-Seq）的超敏感二代测序法检测非小细胞患者的ctDNA中特定的肿瘤基因变异。该方法的主要原理为采用RNA探针捕获技术结合二代测序平台检测基因组变异，由于只需扩增探针捕获的目标DNA序列，且在捕获片段的两端加上接头使每一条DNA片段的3’端和5’端完全相同，因此在测序深度能大大提高的同时保证了扩增的特异性。因此，可有效检测ctDNA中的包括ALK与ROS1融合在内的各种基因突变。

### 2.2 ctDNA的临床应用

在1989年，Stroun等^[83]^首先对肿瘤患者体内的游离循环DNA的序列进行了检测，在肿瘤患者体内的游离循环DNA上相继检测到*K-ras*、*N-ras*、*P53*、*APC*等基因的突变。近年来有较多研究对ctDNA的临床应用进行了探索。但是在循环肿瘤标志物中，ctDNA与CTC因其来源不同，评价标准不同，生物学特征不同，两者可能侧重于不同方面的临床应用（表 2）^[84-86]^。

**2:** CTC和ctDNA临床应用 Clinical application of CTC and ctDNA

Events	Lung cancer screening	Early stage of lung cancer	Advanced lung cancer		Resistance
Therapeutic strategy	Adjutant diagnosis	Recurrence monitoring	Treatment options	Efficacy assessment	Mechanism of resistance, new drugs’ R & D
CTC	√√√	√√√	√√	√√√	√
ctDNA	√√	√	√√√	√	√√√
√: More “√”stands for more clinical application value; ctDNA: circulating tumor DNA.					

#### 2.2.1 肺癌驱动基因检测

由于ctDNA检测基因变异相对快速、便捷，可应用高通量的测序方法，并且部分肿瘤外周血中ctDNA的表达水平高于CTC。因此，对于基因检测方面，虽然两者可互为补充，但可能ctDNA更具一定优势^[87, 88]^。对于*EGFR*突变检测，如上所述的研究已经证实了ctDNA检测*EGFR*突变的可靠性。因此，美国和中国均已有官方批准的血浆检测*EGFR*突变的商业试剂盒。对于*ALK*融合基因检测，目前尚无明确的标准检测方法，有文献报道使用滤膜法联合FISH探针可检测到外周血CTC中的融合基因。针对ctDNA，CAPP-Seq则是较为可行的检测方法之一，但其问题在于DNA层面，融合基因的融合位点往往都在内含子部分，且大部分为随机断裂，因此捕获探针的设计便显得尤为重要。一般而言，由于特异性较高，且有大量文献证实，ctDNA中检出*EGFR*敏感突变可用于指导临床治疗的参考，而检测*ALK*融合基因或者其他驱动基因表达则需要进一步的临床数据来证实。

#### 2.2.2 肺癌辅助诊断

由于ctDNA来源于肿瘤细胞的坏死和凋亡，因此，理论上来说，早期肿瘤外周血中即可能存在ctDNA，但是可能极为微量。二代测序方法与微滴式数字PCR（droplet digital PCR, ddPCR）的敏感度均较ARMS法敏感度高，且可以同时分析多个变异的基因。因此，通过检测ctDNA来辅助诊断肺癌方面，二代测序法具有比较明显的优势。有研究通过CAPP-Seq方法检测13例肺癌患者和5名健康人外周血中的游离DNA，通过检测其中突变或者融合基因来辅助诊断肺癌。研究结果发现总体而言，Ⅱ期-Ⅳ期肺癌与Ⅰ期肺癌的敏感性分别为100%与50%，特异性为96%，对于所有肺癌患者诊断的AUC值为0.89。而且ctDNA的表达量与肿瘤大小相关，相关系数为0.89（*P*=0.000, 2）。由此，研究者认为基于高通量的二代测序技术检测ctDNA中的肿瘤基因变异可用于诊断NSCLC。但是，毕竟ctDNA与CTC不同，循环中游离DNA的来源各异，无法判别来源，且不同时间点游离DNA的表达量亦不相同。另一方面，肺癌的异质性较大，突变谱也较广泛^[89]^，通过ctDNA诊断肺癌的标准尚未统一，是否循环中检测到某种基因变异或者某几类基因变异的组合即可诊断肺癌，目前尚无定论。而对肺癌患者的肿瘤组织和相应的血浆血清中的游离循环DNA进行一些基因启动子区的甲基化检测，发现大多数的肺癌患者都存在一些基因启动子区高甲基化的现象^[90]^。这个方法因此也可以被用来肺癌患者的早期诊断和存活率的检测中。在肺癌患者体内的游离循环DNA中存在杂合性丢失（loss of heterozygosity, LOH）和基因启动子区的甲基化^[91]^，这一发现说明游离循环DNA也许可以用于判断个体是否存在发生肺癌的风险。

#### 2.2.3 靶向药物耐药监测

众所周知，存在驱动基因突变的NSCLC患者需接受相应的靶向治疗。但是，目前的靶向治疗药物，无论是EGFR还是ALK抑制剂均会在10个月左右出现耐药^[7, 92]^，与此同时对于这部分患者耐药的准确判定，实体瘤疗效评价标准（Response Evaluation Criteria in Solid Tumors, RECIST）可能并不完全适用^[93]^。另一方面，酪氨酸结构域的二次突变是导致酪氨酸激酶抑制剂耐药的重要原因之一^[7]^。因此，通过ctDNA检测获得性耐药基因的表达，成为了目前研究的重点方向之一。

2013年，Murtaza等^[94]^在*Nature*杂志上发表了其利用血浆DNA检测观察肿瘤治疗后获得性耐药的研究。其动态观察了6例肿瘤患者（2例乳腺癌、2例卵巢癌、2例肺癌）接受相应治疗后的1年-2年时间中，外周血DNA肿瘤突变数量的变化。结果发现，外周血中突变负荷的增加与治疗的耐药密切相关。其中一例接受吉非替尼治疗的患者在耐药时外周血中检出了T790M突变。在接受EGFR-TKIs治疗的*EGFR*突变患者中，ctDNA中检测T790M突变的技术方法较为普遍。而且，2015年美国临床肿瘤学会（American Society of Clinical Oncology, ASCO）年会中口头报告了ctDNA中T790M突变与三代EGFR-TKIs Rociletinib治疗之间的关系，存在T790M突变患者的有效率为53%，疾病控制率为82%，两者具有明确的相关性^[95]^。2015年发表在*Nature Medicine*中关于三代EGFR-TKIs耐药机制的研究报道，更是提示ctDNA检测在这一方面的前景。该研究通过随访15例T790M突变的患者接受AZD9291治疗，动态监测ctDNA，发现并证实了AZD9291获得性耐药的突变位点C797S^[96]^。并且通过动态观察，发现AZD9291存在三种耐药模式，一种是耐药后T790M突变与C797S突变同时存在，一种是耐药后仅出现T790M的再次升高，第三种是耐药后即不伴有T790M也无C797S的出现。

## 3 小分子RNA（mircoRNA, miRNA）和长链非编码RNA（Long Non-coding RNA）

在生物基因组中广泛存在一类非编码蛋白的RNA基因，其转录物在某种机制下被加工成长度大约20个-24个核苷酸的小RNA，他们因此被命名为miRNA。这类RNA表达谱可以作为某些组织或细胞的特异性分子标志，具有调节其他基因表达的活性，在生物发育过程中发挥着重要的作用。

至今为止，真正确认功能的miRNA还是微乎其微，虽然现在已经找到了1, 000个以上miRNA，并提出他们在细胞增殖、分化、代谢与死亡中发挥着重要的调节作用。Lin-4、Let-7的靶基因是ras信号通路的蛋白^[97-99]^;Lin-41、hbl-1、Lin-14和Lin-28调控时序发育^[100]^等。

长链非编码RNA（long non-coding RNA, lncRNA）是一类本身不编码蛋白、转录本长度超过200 nt的长链非编码RNA分子，他可在多层面上（表观遗传调控、转录调控以及转录后调控等）调控基因的表达。lncRNA最初被认为是RNA聚合酶Ⅱ转录的副产物，是一种“噪音”，不具有生物学功能。然而，近年来的研究表明，lncRNA参与了X染色体沉默、染色体修饰和基因组修饰、转录激活、转录干扰、核内运输等过程，越来越多的人正在研究其调控作用^[101]^。

## 4 小结

通常来说，保证特异性前提下的高敏感度、便于临床应用、可重复性好是CTC与ctDNA检测技术的三个重要评判标准。较高敏感度可保证在数以亿计的背景细胞或者基因组中有效检出CTC或者ctDNA; 便于临床应用是考虑该技术应用于临床的便利性（如检测时间、价格、所需样本大小等）; 可重复性则保证了临床应用的可靠性。因此，从科研探索走向真正的临床应用前，“液态活检”^[102]^的各项检测技术需要通过大规模的临床数据进行验证。否则，只能限制用于科研探索。

CTC和ctDNA仍有不少问题值得我们去探索。关于CTC，外周血中的CTC大部分为具有转移倾向的，对其分子生物学特征的理解有助于我们探索肿瘤发生发展的机制; CTC的富集技术已经日趋成熟，通过高效的富集方法，能否通过病理学手段对循环中的肿瘤细胞进行病理学判定，有助于我们对患者进行真正的无创“液态活检”。关于ctDNA，ctDNA中检测出的基因变异（*EGFR*或者*ALK*）是否可以直接指导临床治疗，我们需要进一步的数据来证实; *EGFR*突变患者使用靶向治疗耐药的过程中，RECIST标准往往评价不够完整，如何通过血液监测耐药突变（T790M）的丰度来指导我们的耐药，也非常值得临床探索。

在将来，随着我们检测方法的不断进步，期待液态活检技术能够真正应用到临床，指导肺癌精准治疗，成为延长患者生存期的必备手段！

## References

[1] Mok TS, Wu YL, Thongprasert S (2009). Gefitinib or carboplatin-paclitaxel in pulmonary adenocarcinoma. N Engl J Med.

[2] Zhou C, Wu YL, Chen G (2011). Erlotinib versus chemotherapy as first-line treatment for patients with advanced EGFR mutation-positive non-small-cell lung cancer (OPTIMAL, CTONG-0802): a multicentre, open-label, randomised, phase 3 study. Lancet Oncol.

[3] Gridelli C, Peters S, Sgambato A (2014). ALK inhibitors in the treatment of advanced NSCLC. Cancer Treat Rev.

[4] Peters S, Adjei AA, Gridelli C (2012). Metastatic non-small-cell lung cancer (NSCLC): ESMO Clinical Practice Guidelines for diagnosis, treatment and follow-up. Ann Oncol.

[5] Zhi XS, Shi YK, Yu JM (2015). China primary lung cancer diagnostic and treatment practices (2015 edition). Zhonghua Zhong Liu Za Zhi.

[6] Sos ML, Rode HB, Heynck S (2010). Chemogenomic profiling provides insights into the limited activity of irreversible EGFR Inhibitors in tumor cells expressing the T790M EGFR resistance mutation. Cancer Res.

[7] Alix-Panabières C, Pantel K (2013). Circulating tumor cells: Liquid biopsy of cancer. Clin Chem.

[8] Hunter K (2006). Host genetics influence tumor metastasis. Nat Rev Cancer.

[9] Maheswaran S, Haher DA (2010). Circulating tumor cells: a window into cancer biology and metastasis. Curr Opin Genet Dev.

[10] Steeg PS (2006). Tumor metastasis: mechanistic insights and clinical challenges. Nat Med.

[11] Dawson SJ, Rosenfeld N, Caldas C (2013). Circulating tumor DNA to monitor metastatic breast cancer. N Engl J Med.

[12] Ashworth TR (1869). A case of cancer in which cells similar to those in the tumors were seen in the blood after death. Aust Med J.

[13] Paget S (1989). The distribution of secondary growths in cancer of the breast. Cancer Metastasis Rev.

[14] Sher YP, Shih JY, Yang PC (2005). Prognosis of non-small cell lung cancer patients by detecting circulating cancer cells in the peripheral blood with multiple marker genes. Clin Cancer Res.

[15] Maheswaran S, Sequist LV, NagrathS (2008). Detection of mutations in *EGFR* in circulating lung-cancer cells. N Engl J Med.

[16] Nieva J, Wendel M, Luttgen MS (2012). High-definition imaging of circulating tumor cells and associated cellular events in non-small cell lung cancer patients: a longitudinal analysis. Phys Biol.

[17] Pantel K, Alix-Panabières C, Riethdorf S (2009). Cancer micrometastases. Nat Rev Clin Oncol.

[18] Stott SL, Lee RJ, Nagrath S (2010). Isolation and characterization of circulating tumor cells from localized and metastatic prostate cancer patients. Sci Transl Med.

[19] Pantel K, Speicher MR (2015). The biology of circulating tumor cells. Oncogene.

[20] Aguirre-Ghiso JA (2007). Models, mechanisms and clinical evidence for cancer dormancy. Nat Rev Cancer.

[21] Barrière G, Tartary M, Rigaud M. (2012). Epithelial mesenchymal transition: a new insight into the detection of circulating tumor cells. ISRN Oncol.

[22] Matrone MA, Whipple RA, Balzer EM (2010). Microtentacles tip the balance of cytoskeletal forces in circulating tumor cells. Cancer Res.

[23] Hou JM, Krebs M, Ward T (2011). Circulating tumor cells as a window on metastasis biology in lung cancer. Am J Pathol.

[24] Hüsemann Y, Geigl JB, Schubert F Systemic spread is an early step in breast cancer. Cancer Cell.

[25] Eyles J, Puaux AL, Wang X (2010). Tumor cells disseminate early, but immunosurveillance limits metastatic outgrowth, in a mouse model of melanoma. J Clin Invest.

[26] He W, Wang H, Hartmann LC (2007). *In vivo* quantitation of rare circulating tumor cells by multiphoton intravital flow cytometry. Proc Natl Acad Sci U S A.

[27] Caceres G, Puskas JA, Magliocco AM (2015). Circulating tumor cells: a window into tumor development and therapeutic effectiveness. Cancer Control.

[28] Sleijfer S, Gratama JW, Sieuwerts AM (2007). Circulating tumor cell detection on its way to routine diagnostic implementation. Eur J Cancer.

[29] Sieuwerts AM1, Kraan J, Bolt J (2009). Anti-epithelial cell adhesion molecule antibodies and the detection of circulating normal-like breast tumor cells. J Natl Cancer Inst.

[30] Königsberg R, Obermayr E, Bises G (2011). Detection of EpCAM positive and negative circulating tumor cells in metastatic breast cancer patients. Acta Oncol.

[31] Liu Z, Jiang M, Zhao J (2007). Circulating tumor cells in periperative esophageal cancer patients: quantitative assay system and potential clinical utility. Clin Cancer Res.

[32] Vona G, Sabile A, Louha M (2000). Isolation by size of epithelial tumor cells: a new method for the immunomorphological and molecular characterization of circulating tumor cells. Am J Pathol.

[33] Gertler R, Rosenberg R, Fuehrer K (2003). Detection of circulating tumor cells in blood using an optimized density gradient centrifugation. Recent Results Cancer Res.

[34] Miller MC, Doyle GV, Terstappen LW (2010). Significance of circulating tumor cells detected by the CellSearch system in patients with metastatic breast colorectal and prostate cancer. J Oncol.

[35] Stott SL, Hsu CH, Tsukrov DI (2010). Isolation of circulating tumor cells using a microvortex-generating herringbone-chip. Proc Natl Acad Sci U S A.

[36] Lou J, Ben S, Yang G (2013). Quantification of rare circulating tumor cells in non-small cell lung cancer by ligand-targeted PCR. PLoS One.

[37] Yu Y, Chen Z, Dong J (2013). Folate receptor-positive circulating tumor cells as a novel diagnostic biomarker in non-small cell lung cancer. Transl Oncol.

[38] Parker N, Turk MJ, Westrick E (2005). Folate receptor expression in carcinomas and normal tissues determined by a quantitative radioligand binding assay. Anal Biochem.

[39] O'Shannessy DJ, Yu G, Smale R (2012). Folate receptor alpha expression in lung cancer: diagnostic and prognostic significance. Oncotarget.

[40] Nunez MI, Behrens C, Woods DM (2012). High expression of folate receptor alpha in lung cancer correlates with adenocarcinoma histology and *EGFR*[corrected] mutation. J Thorac Oncol.

[41] Christoph DC, Asuncion BR, Hassan B (2013). Significance of folate receptor alpha and thymidylate synthase protein expression in patients with non-small-cell lung cancer treated with pemetrexed. J Thorac Oncol.

[42] Cristofanilli M, Budd GT, Ellis MJ (2004). Circulating tumor cells, disease progression, and survival in metastatic breast cancer. N Engl J Med.

[43] Scher HI, Morris MJ, Larson S (2013). Validation and clinical utility of prostate cancer biomarkers. Nat Rev Clin Oncol.

[44] Hayes DF, Cristofanilli M, Budd GT (2006). Circulating tumor cells at each follow-up time point during therapy of metastatic breast cancer patients predict progression-free and overall survival. Clin Cancer Res.

[45] Cohen SJ, Punt CJ, Iannotti N (2008). Relationship of circulating tumor cells to tumor response: progression-free survival, and overall survival in patients with metastatic colorectal cancer. J Clin Oncol.

[46] de Bono JS, Scher HI, Montgomery RB (2008). Circulating tumor cells predict survival benefit from treatment in metastatic castration-resistant prostate cancer. Clin Cancer Res.

[47] Tanaka F, Yoneda K, Kondo N (2009). Circulating tumor cell as a diagnostic marker in primary lung cancer. Clin Cancer Res.

[48] Krebs MG, Sloane R, Priest L (2011). Evaluation and prognostic significance of circulating tumor cells in patients with non-small-cell lung cancer. J Clin Oncol.

[49] Young R, Pailler E, Billiot F (2012). Circulating tumor cells in lung cancer. Acta Cytol.

[50] Sergeant G, Penninckx F, Topal B (2008). Quantitative RT-PCR detection of colorectal tumor cells in peripheral blood: a systematic review. J Surg Res.

[51] Alix-Panabières C, Vendrell JP, Pellé O (2007). Detection and characterization of putative metastatic precursor cells in cancer patients. Clin Chem.

[52] Yu Y, Chen Z, Dong J (2013). Folate receptor-positive circulating tumor cells as a novel diagnostic biomarker in non-small cell lung cancer. Transl Oncol.

[53] Chen X, Zhou F, Li X (2015). Folate receptor-positive circulating tumor cell detected by LT-PCR-Based method as a diagnostic biomarker for non-small-cell lung cancer. J Thorac Oncol.

[54] Krebs MG, Sloane R, Priest L (2011). Evaluation and prognostic significance of circulating tumor cells in patients with non-small-cell lung cancer. J Clin Oncol.

[55] Chen Q, Ge F, Cui W (2013). Lung cancer circulating tumor cells isolated by the EpCAM-independent enrichment strategy correlate with cytokeratin 19-derived CYFRA21-1 and pathological staging. Clin Chim Acta.

[56] Chen YY, Xu GB (2015). Erratum to: Effect of circulating tumor cells combined with negative enrichment and CD45-FISH identification in diagnosis, therapy monitoring and prognosis of primary lung cancer. Med Oncol.

[57] Wu C, Hao H, Li L (2009). Preliminary investigation of the clinical significance of detecting circulating tumor cells enriched from lung cancer patients. J Thorac Oncol.

[58] Wu S, Liu S, Liu Z (2015). Classification of circulating tumor cells by epithelial-mesenchymal transition markers. PLoS One.

[59] Naito T, Tanaka F, Ono A (2012). Prognostic impact of circulating tumor cells in patients with small cell lung cancer. J Thorac Oncol.

[60] Hou JM, Greystoke A, Lancashire L (2009). Evaluation of circulating tumor cells and serological cell death biomarkers in small cell lung cancer patients undergoing chemotherapy. Am J Pathol.

[61] Singletary SE, Greene FL, Sobin LH (2003). Classification of isolated tumor cells: clarification of the 6th edition of the American Joint Committee on Cancer Staging Manual. Cancer.

[62] Sawabata N, Okumura M, Utsumi T (2007). Circulating tumor cells in peripheral blood caused by surgicalmanipulation of non-small-cell lung cancer: pilot study using animmunocytology method. Gen Thorac Cardiovasc Surg.

[63] Barbazán J, Alonso-Alconada L, Muinelo-Romay L (2012). Molecular characterization of circulating tumor cells in human metastatic colorectal cancer. PLoS One.

[64] Liu MC, Shields PG, Warren RD (2009). Circulating tumor cells: a useful predictor of treatment efficacy in metastatic breast cancer. J Clin Oncol.

[65] Punnoose EA, Atwal S, Liu W (2012). Evaluation of circulating tumor cells and circulating tumor DNA in non-small cell lung cancer: association with clinical endpoints in a phase Ⅱ clinical trial of pertuzumab and erlotinib. Clin Cancer Res.

[66] Austrup F, Uciechowski P, Eder C (2000). Prognostic value of genomic alterations in minimal residual cancer cells purified from the blood of breast cancer patients. Br J Cancer.

[67] Soria JC, Gauthier LR, Raymond E (1999). Molecular detection of telomerase-positive circulating epithelial cells in metastatic breast cancer patients. Clin Cancer Res.

[68] Fehm T, Sagalowsky A, Clifford E (2002). Cytogenetic evidence that circulating epithelial cells in patients with carcinoma are malignant. Clin Cancer Res.

[69] Paterlini-Brechot P, Benali NL (2007). Circulating tumor cells (CTC) detection: clinical impact and future directions. Cancer Lett.

[70] Reimers N, Zafrakas K, Assmann V (2004). Expression of extracellular matrix metalloproteases inducer on micrometastatic and primary mammary carcinoma cells. Clin Cancer Res.

[71] Pailler E, Adam J, Barthélémy A (2013). Detection of circulating tumor cells harboring a unique ALK rearrangement in ALK-positive non-small-cell lung cancer. J Clin Oncol.

[72] Jen J, Wu L, Sidransky D (2000). An overview on the isolation and analysis of circulating tumor DNA in plasma and serum. Ann N Y Acad Sci.

[73] Stroun M, Lyautey J, Lederrey C (2001). About the possible origin and mechanism of circulating DNA apoptosis and active DNA release. Clin Chim Acta.

[74] Perkins G, Yap TA, Pope L (2012). Multi-purpose utility of circulating plasma DNA testing in patients with advanced cancers. PLoS One.

[75] Misale S, Yaeger R, Hobor S (2012). Emergence of KRAS mutations and acquired resistance to anti-EGFR therapy in colorectal cancer. Nature.

[76] Chan KC, Jiang P, Zheng YW (2013). Cancer genome scanning in plasma: detection of tumor-associated copy number aberrations, single-nucleotide variants, and tumoral heterogeneity by massively parallel sequencing. Clin Chem.

[77] Yeh CH (2015). Circulating Cell-Free DNA: The blood biopsy in cancer management. MOJ Cell Sci Rep.

[78] Bai H, Mao L, Wang HS (2009). Epidermal growth factor receptor mutations in plasma DNA samples predict tumor response in chinese patients with stages ⅢB to Ⅳ non-small-cell lung cancer. J Clin Oncol.

[79] Han B, Tjulandin S, Hagiwara K (2015). 96O* Determining the prevalence of EGFR mutations in Asian and Russian patients (pts) with advanced non-small-cell lung cancer (ANSCLC) of adenocarcinoma (ADC) and non-ADC histology: IGNITE study. Ann Oncol.

[80] Leary RJ, Sausen M, Kinde I (2012). Detection of chromosomal alterations in the circulation of cancer patients with whole-genome sequencing. Sci Transl Med.

[81] Heitzer E, Ulz P, Belic J (2013). Tumor-associated copy number changes in the circulation of patients with prostate cancer identified through whole-genome sequencing. Genome Med.

[82] Newman AM, Bratman SV, To J (2014). An ultrasensitive method for quantitating circulating tumor DNAwith broad patient coverage. Nat Med.

[83] Stroun M, Anker P, Maurice P (1989). Neoplastic characteristics of the DNA found in the plasma of cancer patients. Oncology.

[84] Vasioukhin V, Anker P, Maurice P (1994). Point mutations of the N-ras gene in the blood plasma DNA of patients with myelodysplastic syndromeor acute myelogenous leukaemia. Br J Haematol.

[85] Sorenson GD, Pribish DM, Valone FH (1994). Soluble normal and mutated DNA sequences from single-copy genes in human blood. Cancer Epidemiol Biomarkers Prev.

[86] Johnson PJ, Lo YM (2002). Plasma nucleic acids in the diagnosis and management of malignant disease. Clin Chem.

[87] Bettegowda C, Sausen M, Leary RJ (2014). Detection of circulating tumor DNA in early-and late-stage human malignancies. Sci Transl Med.

[88] Sausen M, Leary RJ, Jones S (2013). Integrated genomic analyses identify ARID1A and ARID1B alterations in the childhood cancer neuroblastoma. Nat Genet.

[89] Vogelstein B, Papadopoulos N, Velculescu VE (2013). Cancer genome landscapes. Science.

[90] Pan H, Califano J, Ponte JF (2005). Loss of heterozygosity patterns provide fingerprints for genetic heterogeneity in multistep cancer progression of tobacco smoke-induced non-small cell lung cancer. Cancer Res.

[91] Khan S, Coulson JM, Woll PJ (2004). Genetic abnormalities in plasma DNA of patients with lung cancer and other respiratory diseases. Int J Cancer.

[92] Doebele RC, Pilling AB, Aisner DL (2012). Mechanisms of resistance to crizotinib in patients with *ALK* gene rearranged non-small cell lung cancer. Clin Cancer Res.

[93] Choi YL, Soda M, Yamashita Y (2010). *EML4-ALK* mutations in lung cancer that confer resistance to ALK inhibitors. N Engl J Med.

[94] Murtaza M, Dawson SJ, Tsui DW (2013). Non-invasive analysis of acquired resistance to cancer therapy by sequencing of plasma DNA. Nature.

[b95] 95Sequist LV, Goldman JW, Wakelee HA, et al. Efficacy of rociletinib (CO-1686) in plasma-genotyped T790M-positive non-small cell lung cancer (NSCLC) patients (pts). 2015 ASCO, oral abstract 8001.

[96] Thress KS, Paweletz CP, Felip E (2015). Acquired *EGFR* C797S mutation mediates resistance to AZD9291 in non-small cell lung cancer harboring EGFR T790M. Nat Med.

[97] Bartel DP (2004). MicroRNAs: Genomics, biogenesis, mechanism, and function. Cell.

[98] Bartel DP, Chen CZ (2004). Micromanagers of gene expression: The potentially widespread influence of metazoan microRNAs. Nat Rev Genet.

[99] He L, Hannon GJ (2004). MicroRNAs: small RNAs with a big role in gene regulation. Nat Rev Genet.

[100] Lagos-Quintana M, Rauhut R, Meyer J (2003). New microRNAs from mouse and human. RNA.

[101] Wright MW (2014). A short guide to long non-coding RNA gene nomenclature. Hum Genomics.

[102] Haber DA, Velculescu VE (2014). Blood-based analyses of cancer: circulating tumor cells and circulating tumor DNA. Cancer Discov.

[103] Hofman V, Ilie MI, Long E (2011). Detection of circulating tumor cells as a prognostic factor in patients undergoing radical surgery for non-small-cell lung carcinoma: comparison of the efficacy of the CellSearch AssayTM and the isolation by size of epithelial tumor cell method. Int J Cancer.

